# Sex chromosome pre-reduction in male meiosis of *Lethocerus patruelis* (Stål, 1854) (Heteroptera, Belostomatidae) with some notes on the distribution of the species

**DOI:** 10.3897/zookeys.319.4384

**Published:** 2013-07-30

**Authors:** Snejana Grozeva, Valentina G. Kuznetsova, Nikolay Simov, Mario Langourov, Svetla Dalakchieva

**Affiliations:** 1 Institute of Biodiversity and Ecosystem Research, Bulgarian Academy of Sciences, Tsar Osvoboditel Blvd 1, 1000 Sofia, Bulgaria; 2 Zoological Institute, Russian Academy of Sciences, Universitetskaya nab. 1, 199034 St Petersburg, Russia; 3 National Museum of Natural History, Bulgarian Academy of Sciences, Tsar Osvoboditel Blvd 1, 1000 Sofia, Bulgaria

**Keywords:** Karyotype, NOR, meiosis, sex chromosome pre-reduction, male reproductive organs, distribution, *Lethocerus patruelis*, Belostomatidae, Heteroptera

## Abstract

The karyotype and meiosis in males of giant water bug *Lethocerus patruelis* (Heteroptera: Belostomatidae: Lethocerinae) were studied using standard and fluorochrome (CMA_3_ and DAPI) staining of chromosomes. The species was shown to have 2n = 22A + 2m + XY where 2m are a pair of microchromosomes. NORs are located in X and Y chromosomes. Within Belostomatidae, *Lethocerus patruelis* is unique in showing sex chromosome pre-reduction in male meiosis, with the sex chromosomes undergoing reductional division at anaphase I and equational division at anaphase II. Cytogenetic data on the family Belostomatidae are summarized and compared. In addition, the structure of the male internal reproductive organs of *Lethocerus patruelis* is presented, the contemporary distribution of *Lethocerus patruelis* in Bulgaria and in the northern Aegean Islands is discussed, and the first information about the breeding and nymphal development of this species in Bulgaria is provided.

## Introduction

The genus *Lethocerus* Mayr, 1853 is a member of the family Belostomatidae (electric light bugs, toe biters), the subfamily Lethocerinae (Perez Goodwin 2006). The giant water bug *Lethocerus patruelis* is the largest European true bug and the largest European water insect. The adult bugs reach 80 mm in length. The information on the karyotypesof the genus *Lethocerus* has been recently summarized by Bardella et al. (2012). In *Lethocerus* species, chromosome numbers vary from 2n = 4 to 2n = ca. 30 with intermediate numbers of 2n = 8, 26 and 28. The cytogenetic mechanisms of sex determination are also diversified with XY, neo-XY and multiple X_n_Y encountered in different species. In several species, a pair of m-chromosomes (= microchromosomes) has been described (Ueshima 1979). As is common in Belostomatidae and in Heteroptera as a whole, all so far studied species of *Lethocerus* have been shown to have an inverted meiosis for the sex chromosomes in males (the so-called “sex chromosome post-reduction”) with the sex chromosomes undergoing equational separation during the first division while reductional segregation during the second division (Ueshima 1979, Papeschi and Bressa 2006, Bardella et al. 2012).

In the present work, we studied for the first time the structure of the internal reproductive organs, karyotype and meiosis in males of *Lethocerus patruelis* (Stål, 1854). In addition, we summarize here data on the contemporary distribution of *Lethocerus patruelis* in Bulgaria and in the northern Aegean Islands, and provide the first information on the reproduction of this species in Bulgaria.

## Material and methods

### Insects

Males of *Lethocerus patruelis* were collected in 2001–2012 in different regions of southern Bulgaria. Collections were made either in water bodies using plankton net or (predominantly) by light traps. Two adults and three larvae were reared in the laboratory using small fishes (*Gambusia affinis*, *Pseudorasbora parva* and *Carassius gibelio*) as a food. Cytogenetic study was based on three males collected in the area of the border checkpoint Kapitan Andreevo, Bulgaria.

**Specimens examined: BULGARIA: Black Sea Coast**: Burgas, Lukoil Oil Refinery, 29 m a.s.l., May 2011, 4 specimens, K. Popov leg.; **Tundzha River Valley**: Kazanlak, 370 m a.s.l., 15.x.2012, 1♀, Z. Antonova leg.; Elhovo, 113m a.s.l., October 2011, at light, 1♀, G. Hristov leg.; **Maritsa River Valley**: Kapitan Andreevo Checkpoint, 46 m a.s.l.: August–September 2011, at light, more than 60 specimens per night, E. Galabova obs.; 5.ix.2011, at light, 2♂, 4♀, S. Grozeva leg.; 5.x.2011, 4♂, E. Galabova leg.; 12.x.2012, 1♂, at light, E. Galabova leg.; Plovdiv, Plovdiv Thermal Power Station, 170 m a.s.l., October 2011, 1♀ and more than 70 specimens observed in the sewer, Zh. Vlaykov leg; Plovdiv, 168 m a.s.l., autoparking in the northern part of the town, 19.viii.2012, at light, 2 specimens, V. Dimitrov obs.; Peshtera, 435 m a.s.l., October 2011, 1 specimen dead on the road, D. Kajnarov obs.; **Eastern Rhodopes Mts.**: Madzharovo, Vulture Center, above Arda River, 160 m a.s.l., 16.ix.2009, in light trap, 2♂, 1♀, B. Zlatkov leg.; **Kresna Gorge:** above Oshtavska River, 315 m a.s.l., 10.x.2004, at light, 3 specimens, S. Beshkov leg.; Tisata reserve, 250 m a.s.l., 13.x.2012, at light, 1♀, B. Zlatkov & O. Sivilov leg.; **Struma River Valley:** Rupite, 115 m a.s.l., 20.viii.1997, 1 specimen, at light, M. Langourov leg.; Marena artificial pond close to General Todorov Village, 104 m a.s.l.: 28.vi.2009, 1♀, on the vegetation above water surface, V. Gashtarov obs.; 01.viii.2011, 1♀, in the water, M. Langourov leg.; 25.vii.2012, 3 larvae and 2 exuviae, in the water, N. Simov leg.; quarry near General Todorov Village, 113 m a.s.l., 23.x.2010, 1♂, in a puddle near the road, B. Zlatkov & O. Sivilov leg.; **MACEDONIA: Dojran Lake**, 144 m a.s.l., 1996-1997, many specimens, V. Krpach obs.; **GREECE: Thassos Island:** Astris Village, Astris Beach, 7.ix.2011, 1♀, dead on the beach, N. Simov & M. Langourov leg.; Astris Village, small beach N of Astris, 9.ix.2010, 1♀, in the sea, N. Simov & T. Stefanov leg.; Pefkari, 7 m a.s.l., 3.ix.2010, 1♂, dead under street lamps, T. Stefanov leg.

### Preparations

To examine the internal reproductive organs, the abdomen of chloroform-anaesthetized males was opened and the entire reproductive system was dissected. For chromosome studies, the gonads were dissected out from the adults and fixed in Carnoy’s fixative consisting of 96% ethanol and glacial acetic acid (3:1) and stored at 4°C. Cytological preparations were made by squashing a piece of the testis in a drop of 45% acetic acid on a slide. The coverslip was removed using a dry-ice technique (Conger and Fairchild 1953).

### Standard staining of chromosomes

For this staining, the method described in Grozeva et al. (2010) with minor modifications was used. The preparations were first subjected to hydrolysis in 1 N HCl at 60°C for 8 min and stained in Schiff’s reagent for 20 min. After rinsing thoroughly in distilled water, the preparations were additionally stained in 4% Giemsa in Sorensen’s buffer, pH 6.8 for 20 min, rinsed with distilled water, air-dried, and mounted in Entellan.

### Fluorochrome staining of chromosomes

For revealing the base composition of C-heterochromatin, staining by GC-specific chromomycin A_3_ (CMA_3_) and AT- specific 4-6-diamidino-2-phenylindole (DAPI) was used following the method described in Grozeva et al. (2010). C-banding pretreatment was carried out using 0.2 N HCl at room temperature for 30 min, followed by 7-8 min treatment in saturated Ba(OH)_2_ at room temperature and then an incubation in 2xSSC at 60°C for 1 h. The preparations were then stained first with CMA_3_ (0.4 μg/ml) for 25 min and then with DAPI (0.4 μg/ml) for 5 min. After staining, the preparations were rinsed in the McIlvaine buffer, pH 7 and mounted in an antifade medium (700 μl of glycerol, 300 μl of 10 mM McIlvaine buffer, pH 7, and 10 mg of N-propyl gallate).

### Microscopy

The chromosome preparations were examined using the light and fluorescent microscope Axio Scope A1 with digital camera ProgRes MFcool Jenoptik at 100× magnification.

## Results

### Testes

**Figure 1.**
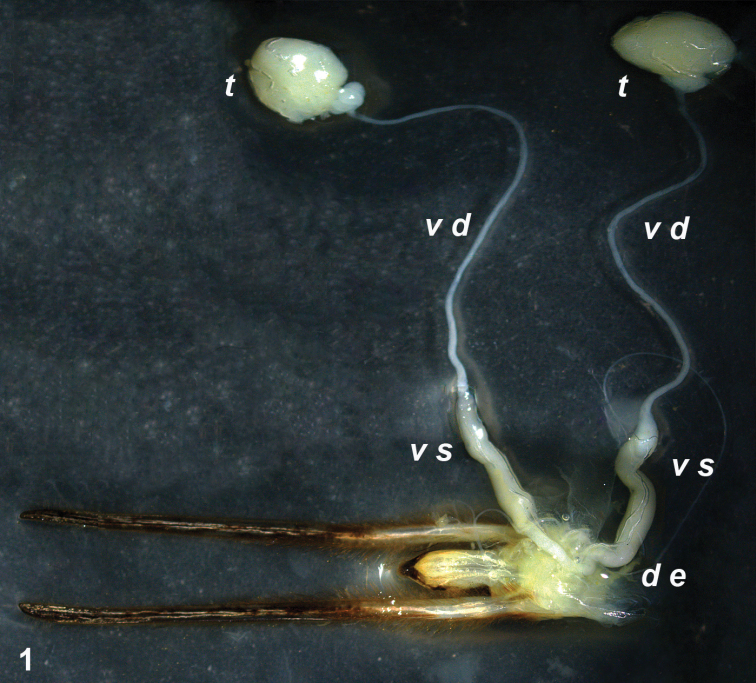
Internal male reproductive system: ***t*** testis; ***v d*** vas deferens; ***v s*** vesicula seminalis ***d e*** ductus ejaculatorius.

In *Lethocerus patruelis* males, the internal reproductive organs consisted of a pair of testes united by vasa deferentia (*v d*) with a median unimpaired tube, ductus ejaculatorius (*d e*) (Fig. 1). Each vas deferens was dilated to form a large vesicula seminalis (*v s*). The testes were colorless and spherical in form, and each consisted of a single very long tube (seminal follicle) rolled up into a ball. The follicle decreased in diameter from the apex to the vas deferens and showed synchronized divisions in different parts, with only sperms in its thinner part. There were no bulbus ejaculatorius and accessory glands.

### Male karyotype and meiosis

**Figure 2–7.**
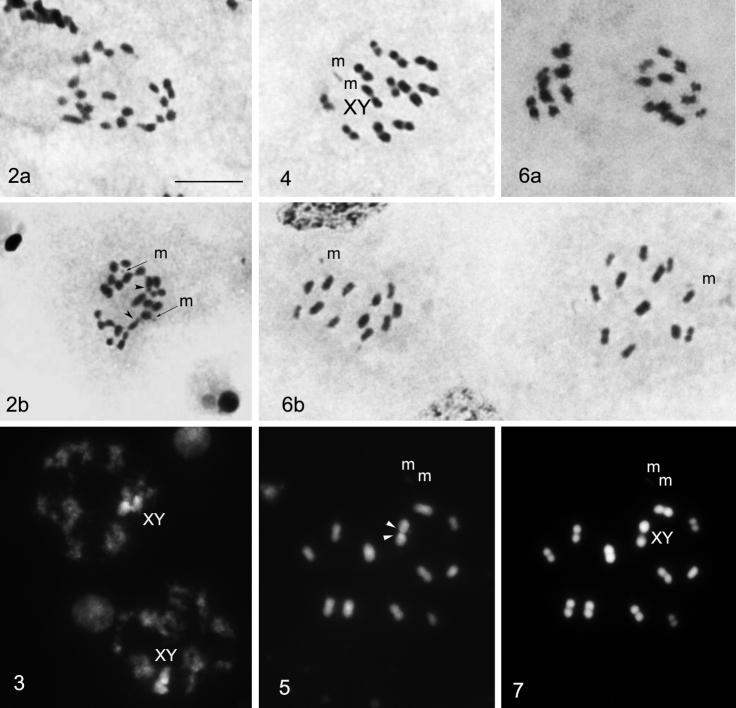
**2** Spermatogonial metaphases: two of larger chromosomes, X and Y, each show a subtelomeric unstained gap, representing the nucleolus organizing region (NOR) (arrow head) (routine staining) **3** Meiotic prophase:sex chromosomes are visible as a large, positively heteropycnotic and brightly fluorescent body (CMA_3_ staining) **4** Metaphase I (n = 13) (routine staining) **5** Metaphase I: GC-rich NORs located on both X and Y chromosomes (CMA_3_ staining) **6** After the first meiotic division all the chromosomes segregate to opposite poles (6a) resulting in two daughter MII cells (6b) with 13 elements each, 11A + m + X and 11A + m + Y, respectively (routine staining) **7** Metaphase I: DAPI staining did not reveal any differentiation along the length of the chromosomes. Bar = 10μm.

All three studied *Lethocerus patruelis* males presented the same chromosome complement. Spermatogonial metaphases showed 26 chromosomes including four larger and two very small ones, and the rest of the chromosomes formed a gradual size row. There was also a pair of very small m-chromosomes (= microchromosomes) (Fig. 2b) but these were not apparent in many nuclei (Fig. 2a). The chromosomes had no primary constrictions, i.e. centromeres. Two of larger chromosomes showed each a subtelomeric unstained gap, or secondary constriction, representing the nucleolus organizing region (NOR). These chromosomes are X and Y sex chromosomes as was revealed by the observation of meiotic stages (see below).

During meiotic prophase, the sex chromosomes were united and visible as a large, positively heteropycnotic body brightly fluorescent after CMA_3_ staining (Fig. 3). Cells at metaphase I (MI) showed 13 bivalents, including a small and negatively heteropycnotic pair of m-chromosomes (n = 13). At this stage, all bivalents were distributed randomly relative to each other. Distinguishing between bivalents of autosomes and XY pseudobivalent involved difficulties since the latter was only slightly heteromorphic in form due to the size resemblance of X and Y chromosomes (Fig. 4). However CMA_3_-staining appeared a foolproof method for the identification of sex-pseudobivalent as one of the largest pairs with GC-rich NORs located in X and Y chromosomes (Fig. 5). At anaphase I (AI), all the chromosomes segregated to opposite poles resulting in two daughter metaphase II (MII) cells with 11A + m + X and 11A + m + Y, respectively (Fig. 6a). In the studied MII plates, X and Y- chromosomes were distributed randomly among other chromosomes (Fig. 6b). DAPI staining did not reveal any differentiation along the length of the chromosomes (Fig. 7).

### Notes to the distribution and reproduction in Bulgaria

In 2008 and 2011, we collected adult specimens of *Lethocerus patruelis* in water bodies in Struma River Valley near Rupite (Bulgaria). Several water bodies in Struma River Valley, close to these localities were checked by plankton net, and in July 2012, four larvae and five exuviae were found in the Marena artificial pond near General Todorov Village representing thus the first evidence of breeding of *Lethocerus patruelis* in Bulgaria. Marena would be classified as semi-natural mesotrophic to eutrophic lake with macrophytic vegetation (Tzonev et al. 2011). The hydrophytic coenoses in Marena make complexes with various hygrophytic communities, e.g. strips or patches of *Typha* spp., *Scirpus lacustris*, tall sedges (*Carex* spp.). Submerged vegetationare mixed by *Myriophyllum* and *Potamogeton*. Larvae were found close to the shoreline, in the regions with submerged vegetation. In laboratory, we observed that larvae and adults had used the stems of *Myriophyllum* as resting place or during stalking/ambush attacks against their preys (electronic supplementary material, video S1).

## Discussion

The range of *Lethocerus patruelis* includes Balkan Peninsula, Anatolia, Israel, Syria, Saudi Arabia, Yemen, the United Arab Emirates, Kuwait, Iraq, Iran, Afghanistan, Oriental Region (Pakistan, India, Nepal, Burma), and recently this species was recorded from Italy (Polhemus 1995, Protić 1998, Perez Goodwyn 2006, Olivieri 2009, Fent et al. 2011).

**Figure 8.**
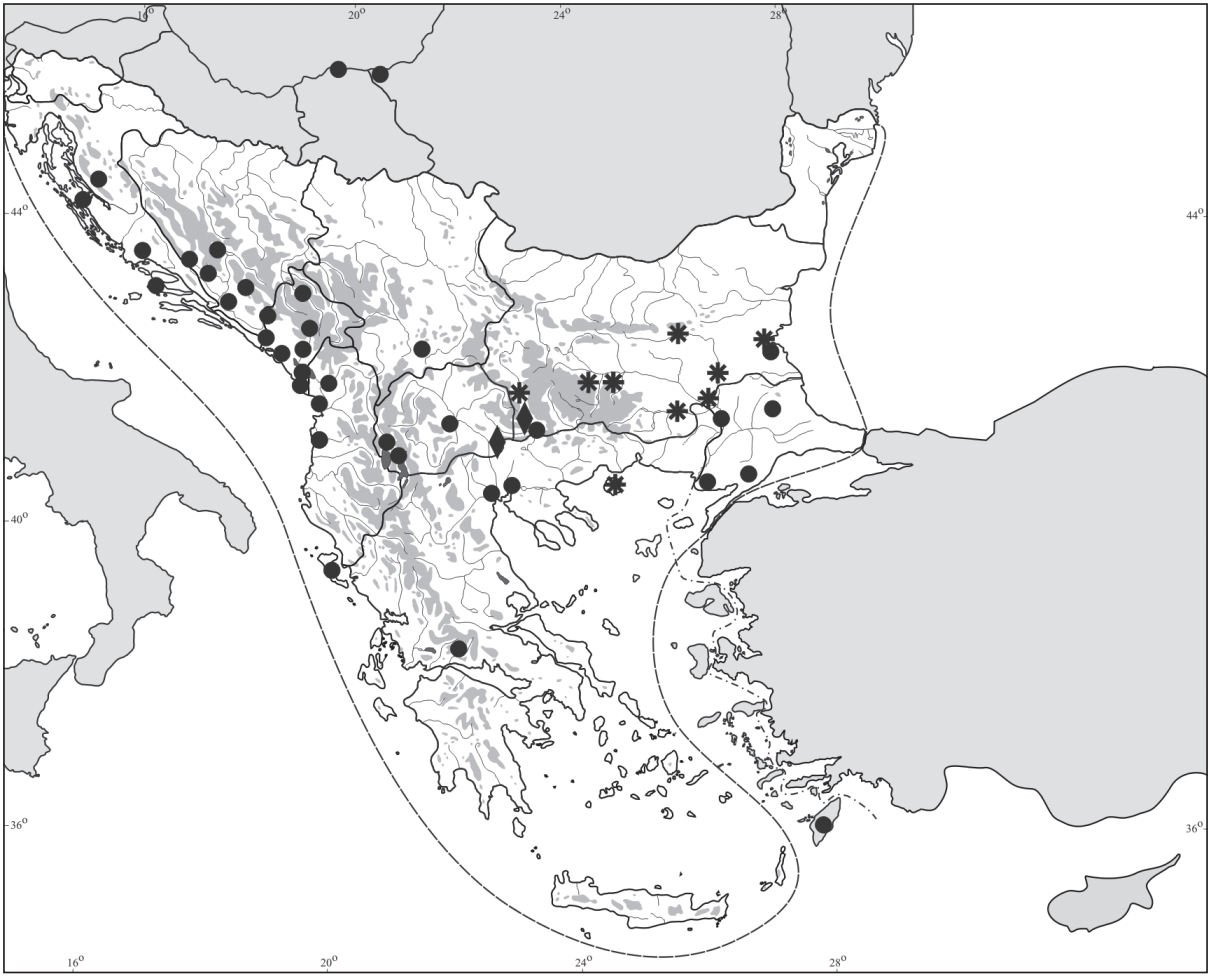
Distribution of *Lethocerus patruelis* (Stål, 1854) on Balkan Peninsula: ● published records; ♦ new records with data of breeding; ✹ new records of specimens attracted to light.

In Bulgaria, only few records of *Lethocerus patruelis* specimens migrating from southern parts of the Balkan Peninsula, attracted to light, were published up to 2000 year (Buresch 1940; Josifov 1960, 1974, 1986, 1999) (Fig. 8). During the last ten years, many new findings of *Lethocerus patruelis* were made by us in Bulgaria: Kresna Gorge, Eastern Rhodopes, Maritsa River Valley (from Kapitan Andreevo to Peshtera) and southern Black Sea Coast (near Burgas). In some of these regions, this species was very abundant; more than 60 specimens per night were attracted to light (Kapitan Andreevo Checkpoint, August–September 2011).

A number of facts (records of the breeding population in Marena; the existence of similar habitats in other regions with records of *Lethocerus patruelis* at light; the last years’ tendency to milder winters) led us to suppose that this species would breed successfully also in other regions in southern Bulgaria (Maritsa River Valley, Burgas lakes). If such is the case, it would be a further evidence of the recent changes of European bug fauna caused by climate changes and global warming (Rabitsch 2008).

We have studied *Lethocerus patruelis* in respect of male reproductive organs, karyotype and meiosis. The internal reproductive system in this species appeared to be quite similar to that in *Diplonychus rusticus* (Fabricius, 1871) (Belostomatinae), the only belostomatid species studied so far on this point (Pendergrast 1957, as *Sphaerodema rusticum*). In *Lethocerus patruelis*, each testis consists of the only follicle which is rolled up into a ball; each vas deferens is dilated to form a large vesicula seminalis; bulbus ejaculatorius (representing usually, if present, a dilated anterior end of the ductus ejaculatorius) and accessory glands (diverticula of the ductus ejaculatorius) are absent. Pendergrast (1957) found a similar condition in *Diplonychus rusticus*, however he did not provide information about the number of follicles in testes.

We found that *Lethocerus patruelis* had 2n = 26 (22 + 2m + XY). The eight *Lethocerus* species studied so far with respect to karyotypes (Table 1) represent a large proportion of the 22 species currently known in this genus (Perez Goodwyn 2006). Hence, some preliminary inferences about cytological features of *Lethocerus* and also of the family Belostomatidae as a whole can be deduced.

Belostomatidae are composed of 3 subfamilies (Belostomatinae, Horvathiniinae, Lethocerinae) with 10 genera and approximately 150 species (Lauck and Menke 1961, Schuh and Slater 1995, Perez Goodwyn 2006, Ribeiro 2007). Up to now, there have been cytogenetically analyzed 32 species of the following 6 genera: *Abedus* (1 species), *Belostoma* (18 species) and *Diplonychus* (3 species) from Belostomatinae and *Benacus* (1 species), *Kirkaldyia* (1 species) and *Lethocerus* (8 species) from Lethocerinae (Table 1). In Belostomatidae, chromosome numbers vary from 2n = 4 in *Lethocerus* sp. from Michigan to 2n = ca. 30 and 2n = 30 in *Lethocerus uhleri* and *Belostoma dilatatum*, respectively. Similarly, the cytogenetic mechanisms of sex determination are variable. Among those, there is the simple XY system (inherent in each of the genera), and the derived neo-XY (in *Lethocerus*) and multiple X_1_X_2_Y or X_1_X_2_X_3_Y (in *Belostoma*) systems. In several species, both from Belostomatinae (*Belostoma* and *Diplonychus*) and Lethocerinae (*Lethocerus*), the presence of m-chromosomes has been reported.

The genus *Lethocerus* shows a fairly wide range of chromosome numbers, with both extreme for Belostomatidae 2n = 4 and 2n = ca. 30, and three intermediate ones of 2n = 8, 26 and 28 (Table 1). The species studied share the conventional cytological features of Heteroptera, such as holokinetic chromosomes (lacking centromeres, that facilitates karyotype evolution via occasional fusion/fission events; Kuznetsova et al. 2011), an XY sex chromosome system (with derivative neo-XY and multiple X_n_Y systems presumed to be inherent in three species), and m-chromosomes (detected to date in *Lethocerus patruelis* and suggestedin *Lethocerus uhleri*, see Table 1). Within the genus, *Lethocerus patruelis* seems to be similar to *Lethocerus indicus* in chromosome complement. This resemblance is confined not only to chromosome number and the presence of m-chromosomes but also to that the sex chromosomes in *Lethocerus patruelis* (present paper) and *Lethocerus indicus* (Bagga 1959, Jande 1959) are indistinguishable from autosomes at meiotic metaphases if a routine staining is used. In *Lethocerus patruelis*, it was due to the size resemblance of X and Y chromosomes causing the almost homomorphic form of the XY-pseudobivalent at MI. Noteworthy however that *Lethocerus indicus* was speculated to have the sex chromosome system of a neo-XY type originated as a result of the evolutionary translocation of both sex chromosomes to one pair of autosomes in the ancestral karyotype (Jande 1959). Another example of a neo-XY system seems to be *Lethocerus* sp. from Michigan. For this species, Chickering and Bacorn (1933) reported 2n = 4 with no distinguishable sex chromosomes. These authors suggested that this karyotype might has originated via a translocation of X and Y chromosomes to one pair of autosomes with a subsequent fusion between two more pairs of autosomes.

**Table 1.** Cytogenetical data for the family Belostomatidae.

**Taxon**	**2n**♂	**Karyotype formula**♂	**Sex chromosome division in male meiosis**	**NOR location**	**Remarks**	**Reference**
**Belostomatinae Leach, 1815**						
*Abedus indentatus* (Haideman, 1854)	29	24 + 2m + X_1_X_2_Y	Post-reduction	data absent		Ueshima 1979
*Belostoma bergi* (Montandon, 1899)	29	26 + X_1_X_2_Y	Post-reduction	data absent		Papeschi and Bressa 2006
*Belostoma bifoveolatum* Spinola, 1852	29	26 + X_1_X_2_Y	Post-reduction	data absent		Papeschi 1991
*Belostoma candidulum* Montandon 1903	16	14 + XY	Post-reduction	sex chromosomes	Technique: CMA_3_ - Bardella et al. 2012	Bardella et al. 2012
*Belostoma cummingsi* De Carlo, 1935	29	26 + X_1_X_2_Y	Post-reduction	sex chromosomes	Technique: Ag-staining, acridine orange, Hoechst 33258 - Papeschi, Bidau 1985. The authors did not specify whether NORs are located on every sex chromosome or only on some of them.	Papeschi and Bidau 1985
*Belostoma dentatum* (Mayr, 1863)	29	26 + X_1_X_2_Y	Post-reduction	a pair of autosomes	Technique: Ag-staining, acridine orange, Hoechst 33258 - Papeschi, Bidau 1985	Papeschi and Bidau 1985
*Belostoma dilatatum* (Dufour, 1863)	29	26 + X_1_X_2_Y	Post-reduction	data absent		Papeschi and Bressa 2006
	30	26 + X_1_X_2_X_3_Y	Post-reduction	the terminal region of a medium-sized autosome pair	Technique: DAPI dull/CMA3-bright band on terminal position on one of the medium-sized autosomal bivalents - Chirino, Bressa 2011;<br/> CMA_3_ - Bardella et al. 2012	Bardella et al. 2012
*Belostoma discretum* Montandon, 1903	29	26 + X_1_X_2_Y	Post-reduction	data absent		Papeschi and Bressa 2006
*Belostoma elegans* (Mayr, 1871)	29	26 + X_1_X_2_Y	Post-reduction	a pair of autosomes	Technique: CMA_3_ - Papeschi, Bidau 1985;<br/> FISH - Papeschi, Bressa 2006	Papeschi and Bidau 1985<br/> Papeschi and Bressa 2006
*Belostoma elongatum* Montandon, 1908	29	26 + X_1_X_2_Y	Post-reduction	data absent		Papeschi and Bressa 2006
*Belostoma flumineum* Say, 1832	24	20 + 2m + XY	Post-reduction	data absent		Chickering 1916, 1927b (after Ueshima 1979)
*Belostoma gestroi* Montandon, 1900	29	26 + X_1_X_2_Y	Post-reduction	data absent		Papeschi and Bressa 2006
*Belostoma martini* (Montandon, 1899)	29	26 + X_1_X_2_Y	Post-reduction	data absent		Papeschi 1991
*Belostoma micantulum* Stål, 1860	16	14 + XY	Post-reduction	sex chromosomes	Technique: CMA_3_, FISH - Papeschi and Bressa 2006	Papeschi 1988
*Belostoma orbiculatum* Estévez & Polhemus, 2001	16	14 + XY	Post-reduction	data absent	Sex chromosome polymorphism (XY/X_1_X_2_Y)	Papeschi 1996: as *Belostoma* sp. (species identification is provided by Papeschi and Bressa 2006)
*Belostoma oxyurum* (Dufour, 1863)	8	6 + XY	Post-reduction	sex chromosomes	Technique: AgNO_3_, acridine orange, Hoechst 33258 - Papeschi and Bidau 1985; CMA_3_, FISH - Papeschi and Bressa 2006	Papeschi and Bidau 1985
*Belostoma plebejum* (Stål, 1860)	16	14 + XY	Post-reduction	data absent	polymorphism for sex chromosomes (XY/X_1_X_2_Y) and number of autosomes (13/14)	Papeschi 1994
*Belostoma* sp.	24	20 + 2m + XY	Post-reduction	data absent		Montgomery 1901, 1906: as Zaitha (after Ueshima 1979)
*Diplonychus annulatus* (Fabricius, 1781)	28	24 + 2m + XY	Post-reduction	data absent		Jande 1959: as *Sphaerodema* (after Ueshima 1979)
*Diplonychus rusticus* (Fabricius, 1781)	28	26 + XY	Post-reduction	data absent		Bawa 1953: as *Sphaerodema*, Jande 1959: as *Spherodema* (after Ueshima 1979)
*Diplonychus molestus* (Dufour, 1863)	28	26 + XY	Post-reduction	data absent		Jande 1959: as *Sphaerodema subrhombeus*, in Ueshima 1979: as *Diplonychus subrhombeus*
**Lethocerinae** Lauck & Menke, 1961						
*Benacus griseus* (Say, 1832)	28	24 + 2m + XY	Post-reduction	data absent		Chickering 1927b (after Ueshima 1979: as *Lethocerus*
*Kirkaldyia deyrolli* (Vuillefroy, 1864)	26	24 + XY	Post-reduction	data absent		Muramoto 1978: as *Lethocerus*
*Lethocerus americanus* (Leidy, 1847)	8	6 + XY	Post-reduction	data absent		Chickering 1927b (after Ueshima 1979)
*Lethocerus annulipes* (Herrich-Schaeffer, 1845)	28	26 + XY	Post-reduction	data absent		Papeschi and Bressa 2006
*Lethocerus indicus* (Lepeletier & Serville, 1825)	26	22 + 2m + XY	Post-reduction	data absent		Banerjee 1958; Bagga 1959; Jande 1959: as neo-XY (after Ueshima 1979)
*Lethocerus melloleitaoi* De Carlo, 1933	28	26 + XY	Post-reduction	data absent		Papeschi and Bressa 2006
*Lethocerus patruelis* (Stål, 1854)	26	22 + 2m + XY	Pre-reduction	sex chromosomes	Technique: CMA_3_	present paper
*Lethocerus uhleri* (Montandon, 1896)	ca. 30	?	Post-reduction	data absent		Chickering and Bacorn 1933: multiple sex chromosomes? (after Ueshima 1979)
*Lethocerus* sp. 1 (from New Orleans)	28	?	Post-reduction	data absent		Chickering 1932 (after Ueshima 1979, n=15)
*Lethocerus* sp. 2 (from Michigan)	4	?	Post-reduction	data absent		Chickering 1927a, 1932; Chickering and Bacorn 1933: as 2 + neoXY: (after Ueshima 1979)

**Figure 9.**
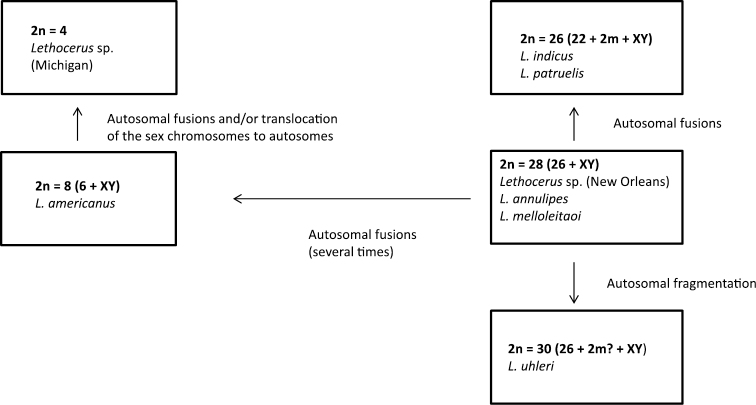
Suggested pathways of karyotype evolution in the genus *Lethocerus*.

Ueshima (1979) considered the karyotype of 2n = 24 + 2m + XY as the modal (the commonest) in the genus *Lethocerus* and the ancestral (i.e., plesiomorphic) one in its evolution. All other karyotypes were suggested to have originated from this ancestral one through autosome fusions and fragmentations, translocations of sex chromosomes to autosomes and loss of m-chromosomes (see Fig. 12 in Ueshima 1979). However, here it should be noted that the most common karyotype needs not to be plesiomorphic in a group (White 1973). In addition, the data available at that time for *Lethocerus* (see Table 4 in Ueshima 1979) were in fact not indicative of the modality of 2n = 24 + 2m + XY in the genus, and some data presented in Ueshima’s scheme were not universally correct (Fig. 9). For example, in the karyotype formulae of some of the species (for instance *Lethocerus* sp. from New Orleans) Ueshima included m-chromosomes which however have not been mentioned in the original paper (Table 1).

On the other hand, the ancestrality of a XY sex determination in *Lethocerus* is beyond question, since neo-XY and X_n_Y systems occurring in Belostomatidae (both), including *Lethocerus* (at least neo-XY), are clearly derivative being originated by X-autosome fusions or X-chromosome fissions, respectively. It cannot be doubted also that low chromosome numbers such as 2n = 8 in *Lethocerus americanus* and 2n = 4 in *Lethocerus* sp. from Michigan, are the derived characters brought about a series of autosome fusions during the course of evolution in this genus.

It seems likely that the ancestral karyotype in *Lethocerus* includes 26 autosomes and XY mechanism as found in many representatives of this genus and Belostomatidae as a whole (Table 1). It is not possible even to suggest whether this karyotype includes a pair of m-chromosomes as was speculated by Ueshima (1979). It is evident that these minute chromosomes easily escape detection by bug cytogeneticists, and hence many species recorded as lacking m-chromosomes in fact have them in their karyotypes.

CMA_3_ staining of *Lethocerus patruelis* C-banded chromosomes revealed GC-rich sites corresponding to NORs in the X and Y chromosomes. This is the first case of NOR detection in *Lethocerus*. On the other hand, ribosomal genes have been already located in *Belostoma* chromosomes using various techniques such as fluorochrome staining, silver staining and FISH (Table 1). In *Belostoma*, five species were shown to have NORs also in sex chromosomes while three other species in a pair of autosomes. Noteworthy, the species with the same chromosome complement sometimes differ in rDNA location (for example, in sex chromosomes in *Belostoma cummingsi* while in autosomes in *Belostoma dentatum*, both with 2n = 26 + X_1_X_2_Y).

In the greatest majority of living organisms, during the first division of meiosis all chromosomes reduce in number (reductional division), whereas during the second division the chromatids separate (equational division), and this pattern is named “pre-reduction” (White 1973). However true bugs characteristically have an inverted sequence of sex chromosome divisions in male meiosis, the so-called sex chromosome “post-reduction” when sex chromosomes undergo equational division at anaphase I and reductional division at anaphase II (Ueshima 1979, Kuznetsova et al. 2011), and this is also true for Belostomatidae (Table 1). Interestingly, *Lethocerus patruelis* appeared unique in showing no inverted sequence of sex chromosome divisions in male meiosis. In this species, X and Y chromosomes form a pseudobivalent at prophase and segregate to opposite poles at anaphase I, and the first division of meiosis is thus reductional both for autosomes and sex chromosomes. As a result of sex chromosome pre-reduction, second spermatocytes carry a single sex chromosome, either X or Y. The second division is then equational for all the chromosomes. Although pre-reduction of sex chromosomes is not usual in Heteroptera, it does occur in some groups (for example, all so far studied species of the family Tingidae have shown pre-reduction; Ueshima 1979, Grozeva and Nokkala 2001). Moreover, closely related species occasionally differ in this pattern (Ueshima 1979, Grozeva et al. 2006, 2007) as is also true of *Lethocerus* species.

Male meiosis in Heteroptera can further be characterized by radial configuration of one or sometimes both MI and MII plates. In this case, autosomal bivalents at MI and autosomes at MII form a ring on the periphery of the spindle, while the sex chromosomes are located in the center of this ring (Ueshima 1979). However in *Lethocerus patruelis*, both MI and MII plates are non-radial with random distribution of all the chromosomes on the spindle.

## Appendix

Electronic supplementary material video S1. (doi: 10.3897/zookeys.319.4384.app) File format: MPEG Video File (mpg).

**Explanation note:** A stalking/ambush attack of *Lethocerus patruelis* larva against small topmouth gudgeon (*Pseudorasbora parva*).
